# GoMapMan: integration, consolidation and visualization of plant gene annotations within the MapMan ontology

**DOI:** 10.1093/nar/gkt1056

**Published:** 2013-11-03

**Authors:** Živa Ramšak, Špela Baebler, Ana Rotter, Matej Korbar, Igor Mozetič, Björn Usadel, Kristina Gruden

**Affiliations:** ^1^Department of Biotechnology and Systems Biology, National Institute of Biology, 1000 Ljubljana, Slovenia, ^2^Department of Knowledge Technologies, Jožef Stefan Institute, 1000 Ljubljana, Slovenia, ^3^Department of Biology, Institute for Biology I, RWTH Aachen University, D-52056 Aachen, Germany and ^4^IBG-2: Plant Sciences, Institute for Bio- and Geosciences, Forschungszentrum Jülich, 52425 Jülich, Germany

## Abstract

GoMapMan (http://www.gomapman.org) is an open web-accessible resource for gene functional annotations in the plant sciences. It was developed to facilitate improvement, consolidation and visualization of gene annotations across several plant species. GoMapMan is based on the MapMan ontology, organized in the form of a hierarchical tree of biological concepts, which describe gene functions. Currently, genes of the model species *Arabidopsis* and three crop species (potato, tomato and rice) are included. The main features of GoMapMan are (i) dynamic and interactive gene product annotation through various curation options; (ii) consolidation of gene annotations for different plant species through the integration of orthologue group information; (iii) traceability of gene ontology changes and annotations; (iv) integration of external knowledge about genes from different public resources; and (v) providing gathered information to high-throughput analysis tools via dynamically generated export files. All of the GoMapMan functionalities are openly available, with the restriction on the curation functions, which require prior registration to ensure traceability of the implemented changes.

## INTRODUCTION

With the development of high-throughput data technologies and consequent rise in the amounts of experimental data available, several controlled vocabularies and ontologies have been developed. In modern plant biology, the most widely used ontologies are the Gene Ontology (GO) (1,2), FunCat (3) and plant-specific Gene Ontology MapMan (4). The MapMan ontology was developed due to the limited scope of prior existing biomedical ontologies for plant-related research, more specifically, to cover plant-specific pathways and processes. It is structured in the form of a hierarchical tree using so-called BINs as ontology concepts (4). BINs describe a wide range of biological terms from the central metabolism to stress responses. As each BIN can be further split into sub-BINs, it allows the ontology flexibility in terms of concept extension and further detailed specification, for example refining concept details related to stress (5) or secondary metabolism (6).

In contrast to hierarchical tree structure of the MapMan ontology, Gene Ontology can be viewed as a collection of three non-overlapping ontologies, namely, molecular function, biological process and cellular component, and the dependencies between concepts in GO are presented in the form of a directed acyclic graph (1,2). The MapMan ontology does not separate between different categories, but the BINs can be separated based on their depth in the ontology, where high-level bins resemble terms in GO biological process ontology and low-level bins are often similar to the terms in the GO molecular function ontology. Therefore, some amount of mapping between both ontologies can be achieved [for detailed comparison of both ontologies, see (7)].

The MapMan ontology was originally built for the model species *Arabidopsis thaliana*, but its aforementioned flexibility allowed for easy transfer of knowledge to other plant species. This resulted in an extension of the ontology to cover several plant species like tomato, potato, Medicago, barley, maize, banana, cotton, grapevine, soybean and tobacco (8). Using the ontology, the MapMan software provides a means to visualize high-throughput data analysis results in a modular manner and in the context of pathways and processes. Although the Gene Ontology and FunCat have been available as a web application for a while, it was not so for the MapMan ontology. Moreover, in the MapMan ontology, the annotations were implemented for each species separately, which can lead to inconsistencies of ontology annotations between different plant species. That, combined with the fact that the web application allows for easier overview and handling of the information, was one of the main motivations for our development of the GoMapMan application and database.

Recent advances in sequencing technologies have tremendously accelerated progress in plant genomics, where genome size can vary by >2300-fold (9). *Arabidopsis* is one of the most studied plant species and its genome sequence has been determined for more than a decade (10). Around 70% of all protein-coding genes of *Arabidopsis* are annotated with either a Gene Ontology or MapMan term (http://www.arabidopsis.org/portals/masc/, Annual Report 2011; http://www.gomapman.org/export/, MapMan export file for *Arabidopsis*). For other plants, with larger genome sizes, multiple whole-genome duplications, segmental duplication events and difficulties in defining accurate gene models (11), the ratio of characterized genes is smaller. This increases the importance of functional knowledge transfer between model plants such as *Arabidopsis* and agronomically important plants. Orthologous proteins (homologous proteins that evolved by speciation only and not by gene duplication) are assumed to retain similar domain architecture and occupy the same functional niche following speciation (12). In this way, the orthologue group information is a valuable source of functional knowledge translation between species, as it allows automatic assignment of function terms to functionally unknown genes from the better-characterized evolutionarily related genes (13). Such propagation of functional knowledge pertaining to phylogenetically related species remains the procedure that scales best and appears most dependable (14) and has been shown to improve the interpretation of large data sets in several studies (15). PLAZA (16), a platform using comparative genomic resources to study gene evolution in plants has been set and is a valuable source of extraction of functional gene relations between species. Additionally, including information from various other sources apart from phylogenetic relationships (for the full list see the next section, ‘Integration’), further improves the task of managing and annotation of novel genes (17). Gene Ontology, for example, uses automatic pipelines to extract gene orthology data, protein sequence signatures and knowledge from external controlled vocabularies to assist in function or location inference (18). To ease the annotation of plant gene functions, the Mercator pipeline for automated sequence annotation was recently developed (Lohse *et al.*, submitted). Such transfer and integration of knowledge is especially valuable for species with limited experimentally derived information, where the sole source of information is Basic Local Alignment Search Tool-based predicted annotations.

**Figure 1.**
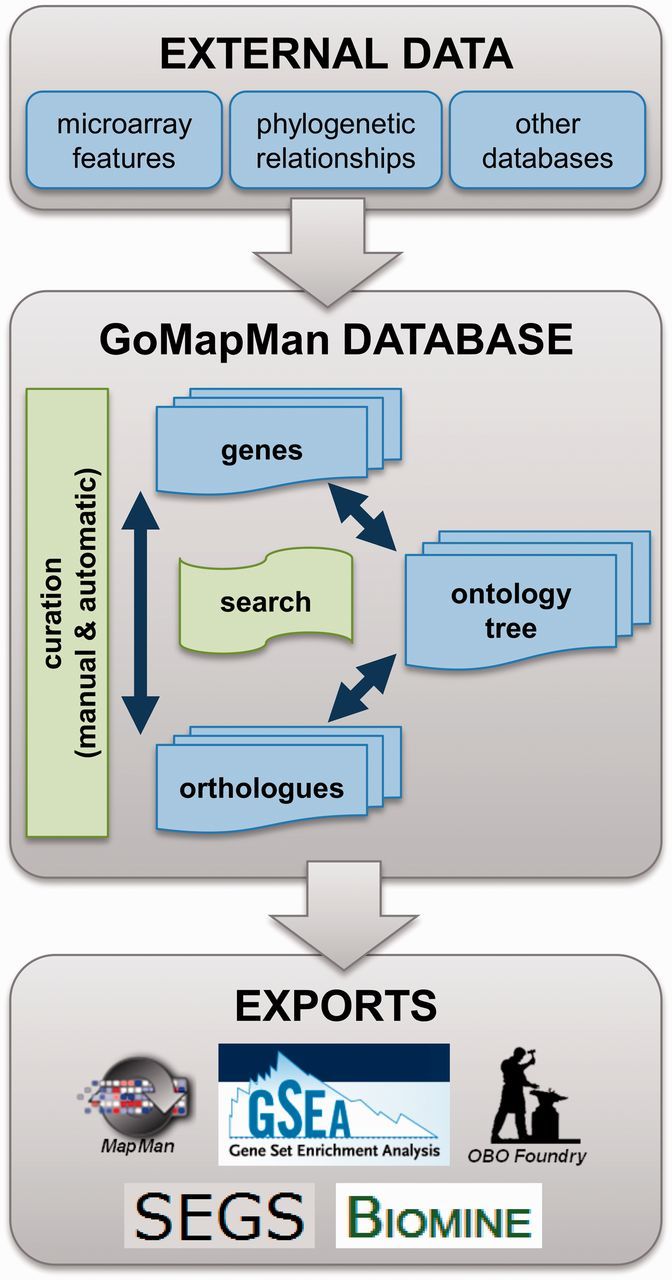
Schematic representation of GoMapMan database functionalities. The data are retrieved from various external sources. Genes are placed into the MapMan ontology tree and connected within a species or between different plant species via orthologue groups. Database contents can be searched or modified by manual or batch curation procedures and are furthermore available in the form of export files for different high-throughput analysis systems.

In this article, we present GoMapMan, a resource for gene functional annotations in the plant sciences (Figure 1). In GoMapMan, genes from selected plant species are organized in the form of an ontology tree, connecting genes from selected species by means of orthologue groups. The information provided by GoMapMan is generated by a combination of automatic procedures in the form of gene annotation retrieval from external databases, automatic consolidation of Gene Ontology annotations through orthologue group information and with additional manual curation (the latter dependant on the expert knowledge of the curators). The gene annotations from the external sources are not validated internally; instead it is left for the user to decide, which information source can be trusted. On the other hand, the ontology information from existing MapMan files available in the MapMan store was consolidated using orthologue information (described in chapter ‘Consolidation’). Traceability of Gene Ontology annotations has been implemented by providing the user a complete history of changes in Gene Ontology annotations. Additionally, dispersed knowledge about genes from several external databases has also been included to provide comprehensive information about the genes. The gathered information can be easily linked to experimental data sets and used by high-throughput analysis tools: different preset export files are dynamically generated to include the current information from the GoMapMan database.

## INTEGRATION: CONNECTING GENES WITH EXPERIMENTAL DATA SETS AND PRIOR KNOWLEDGE

The basic feature of GoMapMan is linking genes of plants included in the database with the MapMan ontology tree. The ontology implemented in GoMapMan was combined from MapMan mapping files for different plant species available at the MapMan website (http://mapman.gabipd.org/web/guest, March, 2012). The reason behind that is that divergent manual curation efforts by communities working on particular plant lead to asynchronous upgrades of the ontology tree. Consequently, different plant species had BINs that were better structured for a particular species. Moreover, the particular BIN may exist only in ontology of one plant and not of the others.

Currently included in GoMapMan database are gene models of the model species *Arabidopsis* and three agriculturally important plant species, rice, tomato and potato. Gene model information was taken from publicly available sources, namely, The Arabidopsis Information Resource (TAIR) for *Arabidopsis* (http://www.arabidopsis.org/), Rice Genome Annotation Project (RGAP) for rice (http://rice.plantbiology.msu.edu/) and Sol Genomics Network for tomato (http://solgenomics.net/). For potato, the gene model situation is complex. Given the high heterozygosity of potato cultivars, the Potato Genome Sequencing Consortium (PGSC) sequenced a doubled monoploid *Solanum tuberosum* line from Group ‘Phureja’ and generated a draft potato genome sequence, on the basis of which two gene models were produced (19,20). As we wanted GoMapMan to contain the information from both potato gene models, as well as information from *Solanum tuberosum* Group ‘Solanum’-derived Unigene sets [namely, Potato Oligo Chip Initiative (POCI) (21) and *Solanum tuberosum* Gene Index (StGI) (22)], that might include potato genes missing in the respective gene models, we have merged all available sequence information into 35 609 gene groups (see Supplementary File S1 for details on the procedure). Each gene group might contain >1 gene (in case of paralogue genes) and is as a rule linked to several identifiers from all four input sources. Novel, so-called *Solanum tuberosum* National Institute of Biology (StNIB) identifiers were generated for these gene groups that match the identifier from one of the input sources. To summarize, 20 809 gene groups are annotated based on the Tomato Genome Consortium (TGC) gene model, 9509 gene groups based on the PGSC gene model, 2882 gene groups based on the POCI Unigene set and 2409 based on the StGI set (the converter table between the newly generated StNIB identifiers and all input gene-related information is provided with the Supplementary File S2).

The genes of plants included in the database can additionally be connected with different microarray platforms (see the complete database schema in Supplementary Figure S1). This GoMapMan feature assists with the biological interpretations of microarray experiments, connecting data with the software that uses ontologies to establish associations between knowledge and data (7). Microarray feature identifiers are in the database stored separately from the gene identifiers, whilst inheriting the ontology annotation from their parental gene identifiers (see Supplementary Figure S1). This allows us to generate exports for different microarray platforms, where microarray feature identifiers are connected to the MapMan ontology. One microarray feature identifier can match several genes and one gene can link to several microarray feature identifiers, due to specificities of microarray design, which is supported by the set-up of the GoMapMan database. Currently, potato feature identifiers for POCI microarray (21) are implemented in the database and mappings for other platforms can be implemented on request and when supplied with an appropriate gene-gene feature identifier mapping file. Nevertheless, the actual analysis of experimental data is left to the user, using the appropriate export formats in tools, such as Gene Set Enrichment Analysis (GSEA) (23) and other supported tools listed in chapter ‘Visualization’.

Another important integration feature of GoMapMan database is accessibility to knowledge stored in different biological databases. These readily accessible data spanning gene functional annotations, protein structure and interactions, pathway information and connections with other ontologies can further improve analyses of high-throughput data and aid in the discovery of previously unknown connections (24) and enable easy knowledge access to researchers interested in a particular gene through browse/search features of GoMapMan. External data in MapMan can be accessed from the *Gene Details View* (described below in chapter ‘Visualization’), where a gene contains links to the external databases, thus enabling easier retrieval of all the relevant information. Currently, existing links to external databases in GoMapMan include Entrez Gene, Gene Ontology, Plant Ontology, PubMed citations, Kyoto Encyclopedia of Genes and Genomes pathways and protein-related information from several databases (EC, Gene3D, HAMAP, InterPro, PANTHER, PFAM, PIR, ProDom, PROSITE, SMART, SPRINT, SUPERFAMILY, TIGRFAM). Collections of external database information, such as BioMart (25) and the connected Ensembl Plants (26), help tremendously, as the need for retrieval from each external database separately is reduced.

*Upgrades of GoMapMan database and updates of data from external sources*. GoMapMan has been designed to expand and improve—to include new plant species, to improve Gene Ontology and gene annotations and to dynamically upgrade other types of information contained in the database. Large modifications to the database are implemented in a form of modular batch scripts. These scripts serve several purposes: (i) the addition of new plant species; (ii) orthologue-related modifications; (iii) insertion/retrieval of additional microarray feature identifiers of various microarray designs; and (iv) insertion/retrieval of annotations from external databases. All batch scripts have a predetermined input format, which makes the tracking of changes easier and makes the import into the database quicker. The clear separation of tasks that the scripts perform and their inherent modularity also give a lot of potential for development of other specialized features of GoMapMan.

## CONSOLIDATING ONTOLOGY ANNOTATIONS FOR DIFFERENT PLANT SPECIES

Another key motivation in the creation of the GoMapMan application was to enable propagation of functional knowledge from evolutionary relationships, based on the fact that similar genes often have conserved functions in different organisms (27). GoMapMan uses orthologue group information as its main source of functional knowledge translation between species, so-called consolidation of annotations between species.

In GoMapMan, the orthologous group information is derived from public sources and the database has been designed to allow for several orthologous groupings to be included. Orthology detection methods differ in their sensitivity and specificity (28) and orthologue detection varies depending on data features and their taxonomic context (29,30). For that reason, GoMapMan can include orthologous relationships with broader or smaller taxonomical ranges, which result in lower or fine-grained orthologous groupings, respectively. Inclusion of orthologue information allows one to observe genes of several plant species at the same time, allows synchronous management of ontology annotations for several genes at the same time and enables the use of automatic Gene Ontology annotation consolidation scripts. Currently, two orthologue groupings are included in GoMapMan. The first was performed on proteomes of 12 plant species by the PGSC (19) using the orthoMCL algorithm (31). The second orthologue grouping was provided by The International Tomato Annotation Group using reciprocal-smallest distance method (32) that includes four plant species (*Arabidopsis*, potato, tomato and grapevine).

Over 60% of all protein-coding genes for the two members of the Solanaceae species (tomato, potato) are included in at least one orthologue grouping (Table 1). High percentages of intra and inter-species orthologue clusters enable efficient improvement of functional annotations. As one orthologue group can contain several genes of one plant species, it also improves ontology annotations within a single species, useful in the plant sciences particularly, where some gene families can be large.

**Table 1.** Number of protein-coding genes for each species and percentage of genes included in any of the orthologue groups

Species	Total number of protein-coding genes	Number of protein-coding genes present in at least one orthologue group
*Arabidopsis*	27 416	23 446 (86%)
Tomato	34 732	22 186 (64%)
Potato	35 609	23 882 (67%)
Rice	39 045	29 183 (74%)

**Figure 2.**
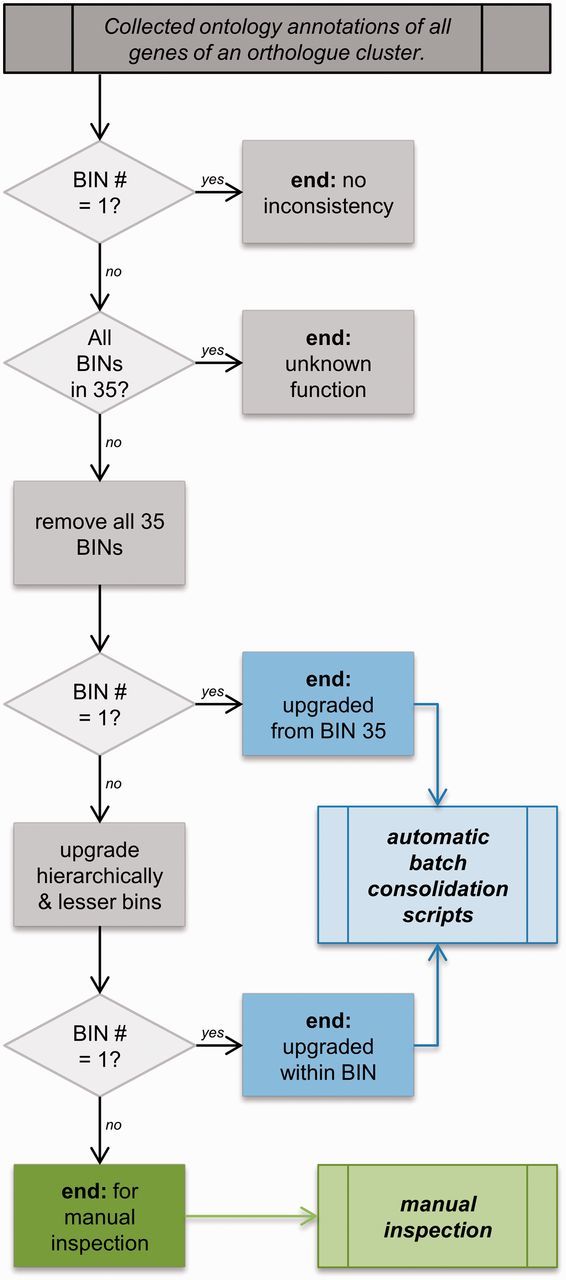
Consolidation scheme for improvement of Gene Ontology annotations as implemented in GoMapMan. Consolidation enables transfer of knowledge across plant species through the use of orthologous gene groups. In the first step for each orthologue group, the ontology positions of genes contained are gathered. If the unique BIN count equals 1, then the genes of the orthologue group have ‘no inconsistencies’ and no consolidation is performed. If all the BINs are marked as unknown, not annotated or previously not described (assigned to BIN 35), genes have an ‘unknown function’ and remain in BIN 35. If at least one of the ontology positions does not equal BIN 35, then genes are deleted from BIN 35. If the number of remaining unique BINs after this procedure equals 1, then they are ‘upgraded from 35’ and a list for semi-automatic batch scripts is generated. The remaining gene groups are then in the last part upgraded into lower fine-grained hierarchical levels, and BINs described as miscellaneous are removed and if the remaining BIN count equals 1 they are considered to be *upgraded* ‘within the BIN’ if the BIN count equals 1. The remaining orthologue group requires a manual check by an expert (green block).

We have implemented a set of custom built consolidation scripts (Figure 2) that run internally and first gather ontology annotations from genes belonging to an orthologue group. This is then followed by a set of rules, which check whether any gene from the cluster can be upgraded with respect to its ontology annotation and ontology annotation of other genes in the cluster. There are two outcome types of these condition blocks: the first a list of tasks for the automatic batch curation scripts (described in subsection Curation) and the second a list of genes for manual inspection by a human expert (Figure 2, light blue and green blocks, respectively). Consolidation scripts are run in the background, whenever a new orthologue type or plant species is added into the database.

A comparison between the most recent potato POCI microarray export available at GoMapMan (http://www.gomapman.org/export/current, POCI_Agi_mapping, 16-Jul-2013) with an older version available at MapMan store (http://mapman.gabipd.org/web/guest/mapmanstore, Potato_POCI, Sep08) showed that about half of the identifiers that were once in BINs 35, which includes unknown, not annotated or previously not described genes, were moved to other BINs using our consolidation procedure.

## CURATION: DYNAMIC AND INTERACTIVE GENE PRODUCT ANNOTATION

**Figure 3.**
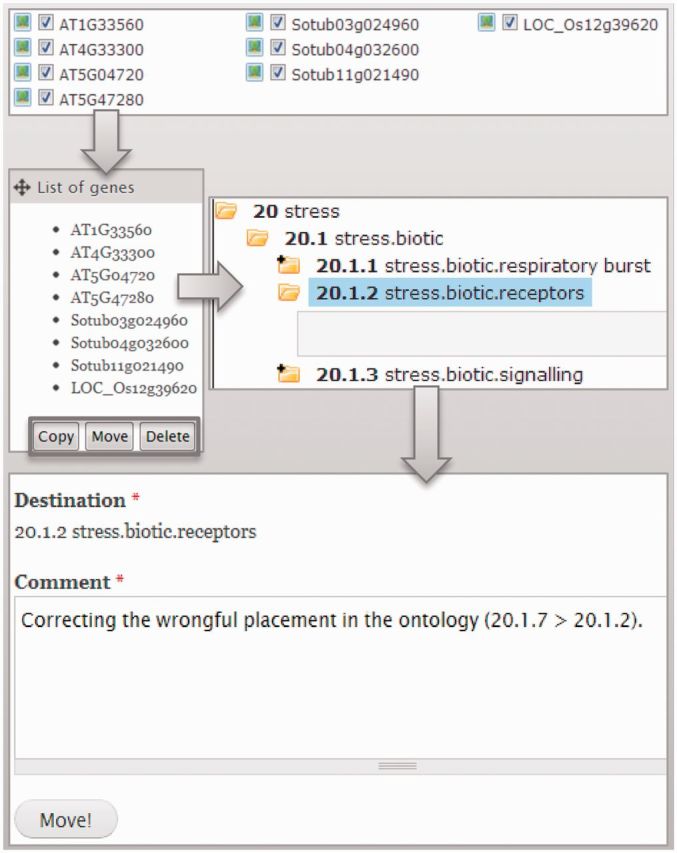
Manual curation of gene annotations—to assist the experts in manual curation of the Gene Ontology annotations, a simple interactive feature was implemented. It is a four step procedure, which starts with the user selecting the genes to be modified. These selected genes are then listed, and an appropriate action has to be selected, depending on the desired modification. With the exception of a deletion from the BIN, the BIN selection window with the comment box opens. On successful modification of the database, the user is additionally informed.

GoMapMan has been set up in a way that makes the process of curation simpler for interested parties in the scientific community (Figure 3). Access to these tools is limited to authorized users, usually domain experts, who would wish to contribute to the expansion and improvement of the knowledge contained in the database (registration information available on the web page).

Several approaches for curation are available through GoMapMan application:

*Ontology tree curation*. New knowledge about the biological pathways and processes can be easily transferred to the ontology tree. As the MapMan ontology has been designed with flexibility in mind, the ontology tree can be easily branched out further. For example, the BIN for secondary metabolism of phenylpropanoids (BIN 16.2 secondary metabolism phenylpropanoids) only contains the lignin biosynthesis (BIN 16.2.1) and unspecified assignments (BIN 16.2.99). A new sub-BIN, related to phenylpropanoids degradation, can be added either as the next number in the series (BIN 16.2.2), or an arbitrarily selected value, as long as it is not in use already (BIN 16.2.20). The ontology tree curation can be done only manually, as we do not expect ontology tree modifications to be needed often or on a large scale. A script enabling ontology tree curation with a predetermined input format is part of GoMapMan application.

*Interactive manual curation of gene annotations*. To change incorrect automatic annotations or just to annotate a gene with a newly determined function, manual corrections and human supervision of the annotation process are enabled in GoMapMan via the graphical user interface (Figure 3). Several genes can be selected and either copied (if additional function was discovered), moved into other bins (if the original annotation was incorrect and a new function can be assigned to the gene) or deleted (if the original annotation for the gene was wrong). Specified action request is transferred to background scripts that check the validity of the action (e.g. errors will be given if we want to assign an already existing ontology annotation to a gene) and perform the actions if there are no issues.

*Batch manual curation of gene annotations*. For the larger scale modifications of gene annotations a batch script was prepared that runs on predetermined input format. The biological expert fills in the input file with the novel information on gene annotations and the batch script is later run by the administrator to implement the changes.

*Automatic modifications of gene annotations with the help of consolidation scripts*. As described earlier in the text, gene annotations are improved across all plants in the database whenever a new orthologue type or plant species is added. Similarly, all changes of gene annotations within one species (performed in interactive manual curation mode) are transferred also to other plants through the use of consolidation scripts that are run at least quarterly.

Traceability of gene ontology annotations has been implemented with all levels of curation to give the user an idea of gene history in terms of the MapMan ontology positions and authorship to curation efforts.

## VISUALIZATION OF DATA AND KNOWLEDGE WITHIN GoMapMan

**Figure 4.**
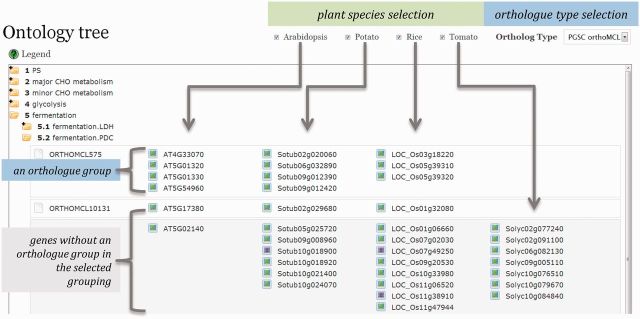
Ontology Tree view in GoMapMan. The BINs (denoted with a yellow folder icon) are displayed as a tree, one BIN can be viewed at a time. On top the user can select the plants that will be displayed and select the orthologue type from a drop-down menu. Depending on the orthologoue type selected, the display is dynamically updated. The orthologue groups are listed with their corresponding genes and the genes that do not have an orthologue in the current orthologue type selection are listed at the end.

*Ontology Tree view*. The main view in GoMapMan is the Ontology tree as displayed in Figure 4. The hierarchically organized MapMan BINs are displayed as opened or closed folders, and the latter has a plus symbol, indicating that it contains further sub-BINs. Each BIN further contains orthologues and plant genes, where species of the displayed genes can be selected by the user. To improve readability, only one orthologue grouping can be viewed at a time. The colouring of each gene’s icon indicates whether or not it belongs to any orthologue groups implemented in the database, the green indicating belonging to at least one and the purple indicating genes not in any.

Clicking any of the gene or orthologue identifiers opens into two new views, the gene details view and orthologue details view, respectively.

*Gene details view*. This view lists gene details of a unique gene identifier that is linked out to the parental database of that plant (e.g. TAIR for *Arabidopsis*). This enables easier search for additional annotations and information contained in the parental database. Gene descriptions, also derived from the original source, are displayed within gene details view. Where available, we list the short names, synonyms and genomic context related to the gene. Also listed is the source, which represents the gene model version (e.g. TAIR9). In the following sections of the gene details view additional gene information is listed: (i) ‘ontology annotations’—all the MapMan BINs the gene is annotated with; (ii) ‘orthologues’—the orthologue groups the gene is present in; (iii) ‘microarray features’—links of gene with different sets of microarray feature identifiers; and (iv) ‘external annotations’—table with information from different relevant external knowledge resources with provided links.

*Orthologue details view*. Orthologue details view is organized similarly to the gene details view. The first section contains the orthologue cluster identifier and the type (short description of the source and algorithm used to produce the orthologue groups). The ‘Ontology annotations’ connect the orthologue cluster with relevant MapMan BIN(s) and ‘Orthologue genes’ sections list cluster genes of plant species that are supported by GoMapMan (currently *Arabidopsis*, rice, potato and tomato). As the orthologue group analysis included in GoMapMan was performed on additional plant species that GoMapMan does not support currently, the genes of the remaining plants are displayed as ‘Annotations in Orthologue Details view’. Their gene identifiers are connecting to an external database to provide access to additional knowledge if needed. Such structure simplifies the addition of new plant species into GoMapMan, as the genes of the newly added plant inherit the MapMan annotations from the genes we already contained in the database before the inclusion of a new plant species gene model.

*Search tab view*. Simple search options are implemented in GoMapMan. The user can select the search to be performed on genes, orthologues or microarray feature identifiers. Additionally, for gene and microarray features the user can limit the search to only the desired species. The results are then listed on the same main page, the list of genes that meet the search criteria, short gene names and MapManBIN codes the gene is annotated with. When searching for microarray feature identifiers, the results returned are actually gene identifiers.

*Exports tab view*. The curation and consolidation efforts lead to improvements of the ontology assignments that can be used in different high-throughput analyses tools. Within GoMapMan this is dynamically enabled through generation of different export files. The tools the exports have been designed for are MapMan, GSEA, search for enriched gene sets (SEGS), Biomine and SegMine. A directory containing the data from current and previous versions of ‘exports’ files can be accessed from GoMapMan main page. Updates of the export files are planned for any major revision of the database (such as an addition of a new plant, changes in the MapMan ontology or batch curations of Gene annotations) or at least every 3 months.

- *Generic*. This section contains some database exports in a neutral format (ontology export; connections between orthologue clusters and gene identifiers for each orthologue grouping; list of genes and BIN codes for each plant; connections between gene and microarray feature identifiers).
- *The Open Biological and Biomedical Ontologies (OBO)*. The export of the ontology in an OBO (33) concordant format is contained in this folder.
- *MapMan*. To display the experimental results onto diagrams of metabolic pathways or other processes, MapMan software (4) requires so-called mapping files. For each plant, gene or microarray feature identifiers with their inherited ontology annotations are exported in a format suitable for import to MapMan software.
- *GSEA*. Genes or microarray feature identifiers for plants with their ontology annotations are exported in the gene matrix transposed file format (*.gmt), which is required by the GSEA software (23).
- *SEGS*. Alternative enrichment analyses can be done by SEGS (34). Exports require a transposed form of GSEA format: each row belongs to a plant gene or microarray feature identifier followed by a list of all MapMan BINs it is present in.
- *Biomine*. Biomine integrates data from several biological databases into a common graph model, whose goal is to enable discovery of new connections between entities (24). Biomine export structure is composed of separate files for each plant and type of link relation. These files can be imported into the Biomine database and used by powerful graph search algorithms to support discoveries of new links.
- *SegMine*. SegMine methodoloy has been developed for semantic analysis of microarray data and exploits general biological knowledge in a new workflow environment, Orange4WS (35). By combining the export files for Biomine and SEGS, the use of SegMine application is enabled as well for plants.

## GoMapMan TECHNICAL SPECIFICATIONS

GoMapMan homepage: http://www.gomapman.org

Contact: gomapman@nib.si; gomapman@ijs.si

GoMapMan is hosted on an Apache Hyper-Text Transfer Protocol (HTTP) server with Fedora operating system. The data are stored and managed using the MySQL relational database management system combined with a dynamic web interface that was built in PHP, JavaScript and Drupal. GoMapMan website is fully functional in all major browsers with enabled JavaScript. There are no access restrictions for academic and commercial use, and we encourage the active involvement of interested researchers, be it by sending us a request for changes or requesting registered access to the database.

## CONCLUSIONS

To conclude, GoMapMan is set to assist plant research community in the era of tremendous experimental data expansion. Its flexibility enables easy expansions to include additional plant species in one dimension and improve gene annotations according to current knowledge in the other. Implementation of orthologue groups connects both dimensions for efficient transfer of knowledge from one species to another. To better support GoMapMan for systems biology-oriented research, we also plan to add other cell components to MapMan ontology besides genes. Metabolite MapMan ontology already exists and requires a simple addition into the database. Similarly, ontology for the microRNAs (now all listed in BIN32) can be implemented according to their targets ontologies. Integration of knowledge from other public resources and preset export system for different high-throughput data analysis application is suitable for easy access of information about single genes as well as for biological interpretation of whole-genome datasets. Taken altogether, we believe that GoMapMan will bring additional insights into different areas of plant biology research and promote future synergies between research groups.

## SUPPLEMENTARY DATA

Supplementary Data are available at NAR online, including [36–42].

## FUNDING

Slovenian Research Agency (ARRS) [J4-4165, J2-2353, P2-0103, P4-0165]. Funding for open access charge: Slovenian Research Agency (ARRS) [J4-4165].

*Conflict of interest statement*. None declared.
